# “It’s Like a Whole Set of Things That We Do”: A Qualitative Study of the Roles of Journal Editors-in-Chief

**DOI:** 10.5334/pme.3277

**Published:** 2026-10-15

**Authors:** Lauren A. Maggio, Anthony R. Artino, Joseph A. Costello, Paolo C. Martin, TingLan Ma, Kirsten R. Brown

**Affiliations:** 1 Department of Medical Education, University of Illinois College of Medicine, Chicago, IL, US; 2 Department of Health, Human Function, and Rehabilitation Sciences, George Washington University School of Medicine and Health Sciences, Washington, DC, US; 3 University of Illinois College of Medicine, Chicago, Illinois, US; 4 Department of Health Professions Education, Uniformed Services University of the Health Sciences, Bethesda, MD, US; 5 Department of Health Professions Education, Uniformed Services University of the Health Sciences, Bethesda, MD, US

## Abstract

**Introduction:**

Journal editors-in-chief (EICs) play a central role in shaping scholarly publishing, yet little is known about how they understand and enact their roles. We explored how medical education journal EICs describe their roles in shaping the publication process.

**Methods:**

We conducted a qualitative study using thematic analysis of interviews with nine medical education journal EICs. The interviews were part of a broader investigation of publication timelines and explored editorial leadership and practices, including experiences during the COVID-19 pandemic. Participants’ accounts also provided insight into the broader work of leading a journal, which became the focus of the present analysis.

**Results:**

Participants described their work not as a set of discrete tasks, but as five interrelated roles: *knowledge developer, proactive communicator, steward of time and energy, nurturer*, and *orchestrator*. These roles were enacted simultaneously and often in tension, reflecting the need to balance scholarly rigor, relational commitments, and system constraints. EICs described actively shaping knowledge, orchestrating complex communication networks, safeguarding finite reviewer and editorial resources, supporting authors and editorial teams, and maintaining workflow continuity. Much of this work was characterized as invisible labor occurring behind the scenes and not captured in formal metrics. Participants also highlighted a structural tension between nurturing authors and stewarding limited resources, underscoring the challenges of sustaining both inclusivity and efficiency within a resource-constrained system.

**Discussion:**

This study offers a practice-informed framework for understanding EIC work as relational, strategic, and system-oriented. By making visible the often-invisible labor that underpins academic publishing, our findings challenge reductive conceptions of editorial work and highlight the need for training, support, and systemic changes to better align expectations for rigor, inclusivity, and sustainability.

## Introduction

Publishing in academic journals is a high-stakes process, shaping hiring, promotion, and career advancement of scholars [1]. Yet, for many authors, the publication process remains opaque, often described as a “black box,” [2 (p1)] thereby contributing to author uncertainty and frustration with journals and their editors [3,4,5]. In response, scholars have examined measurable features of the publication process. For example, quantitative studies have explored publication timelines [6,7,8], the composition of editorial teams [9,10], and peer-review outcomes [11,12]. While these quantitative analyses offer useful benchmarks and support transparency, they provide limited insight into the human and organizational processes through which publishing occurs.

Central to the publishing process is the editor-in-chief (EIC), a pivotal, yet underexamined role in academic publishing [13]. EICs operate at the intersection of authors, reviewers, editorial teams, and publishers, holding responsibility for peer-review oversight, editorial decision making, and journal direction. The breadth of EIC responsibilities is summarized in a 14-point competency-based framework that ranges from demonstrating knowledge of their field to evaluating the scientific integrity and rigor of manuscripts [14]. Although these descriptions convey the scope of editorial responsibility, they offer limited insight into how EICs conceptualize and enact these roles in practice.

Empirical accounts of EICs’ lived experiences remain scarce. Much of what is known about editorial work is inferred from EICs’ individual reflections [2,15], calls for applications [16], or survey-based studies [17,18]. Few descriptions are grounded in EICs’ collective accounts, with existing qualitative studies only addressing specific facets of the role (e.g., gatekeepers, developers) [13]. In biomedicine, qualitative studies have focused on EICs’ perspectives on peer reviewers and the review process, rather than their own roles [19]. In medical education, Cheung and colleagues examined how EICs describe their roles in capacity building, highlighting efforts to “make space” for diverse authors and perspectives [20 (p1308)]. Although informative, these studies do not capture a holistic picture of the human, relational, and organizational work of leading a journal.

This gap constrains how we understand academic publishing. Without attention to how EICs navigate daily realities, key features of the publishing process risk being interpreted as static outputs rather than the product of complex, socially organized work. As academic publishing increasingly emphasizes transparency, efficiency, and equity [21,22], a more nuanced understanding of EICs’ roles is essential to contextualizing how journals function and how knowledge is produced and legitimized. Such transparency may also benefit authors by enabling more informed planning and reducing uncertainty in navigating the publication process.

This study draws on interviews conducted as part of a broader investigation of publication timelines and editorial experiences during the COVID-19 pandemic. We explore how medical education journal EICs understand and enact their roles in shaping the publication process. By examining EICs’ accounts of their editorial responsibilities, this study aims to provide a richer, practice-informed understanding of the human and organizational work through which scholarly publishing occurs.

## Methods

We conducted a qualitative study using reflexive thematic analysis [23,24], guided by a constructivist paradigm. The interviews analyzed in this study were originally conducted as part of a larger, multi-methods investigation of publication timelines in health professions education (HPE) publishing, which included both author and EIC participants representing journals that publish in English. Findings from the quantitative component [6] raised important questions about the influence of editorial processes and roles on the publication process, prompting an interpretive qualitative inquiry designed to extend and contextualize those findings through EICs’ accounts. The EIC interviews explored publication timelines and editorial experiences, with substantial attention to the disruptions and adaptations associated with the COVID-19 pandemic. During our engagement with these data, participants’ accounts repeatedly extended beyond timelines and pandemic-specific experiences to describe the broader work they did to lead journals and sustain the publication process. These accounts became the focus of the present analysis.

We identified EICs, as of January 2024, from medical education journals included in the MEJ-24 (Medical Education Journals). The MEJ-24 is a bibliometrically derived set of journals intended to represent the core medical education literature [25]. Journal mastheads were used to identify eligible participants, yielding a sample frame of 22 EICs. One journal, *BMJ Simulation & Technology Enhanced Learning*, had ceased publication, and *BMC Medical Education* does not make its EIC’s identity public. The Uniformed Services University Human Research Protection Program Office declared this study exempt (967740).

LM recruited participants via email. Given the specificity of our participant group and the richness we observed in our interview data, we considered the nine interviews we conducted to provide sufficient information power for this analysis [26,27].

We developed a semi-structured interview guide informed by the literature and our experiences as editors (LM and AA) and authors in medical education journals. See the supplementary materials for the interview guide. The guide was developed for the broader study and focused primarily on publication timelines, editorial leadership, and experiences and adaptations during the COVID-19 pandemic. Its semi-structured format also allowed participants to elaborate on their editorial practices and responsibilities. Questions about editorial leadership, challenges and adaptations, communication, and managing publication timelines were particularly helpful in eliciting broader accounts of participants’ editorial practices and responsibilities. The interview guide was piloted with an EIC of a journal not included in the MEJ-24, but that publishes HPE-related articles, and was refined based on pilot feedback.

LM conducted all interviews between January and April 2024. At the time of data collection, LM was the Deputy EIC of *Perspectives on Medical Education* and had previously held multiple editorial roles in HPE journals, including associate editor and EIC roles. LM’s editorial background was leveraged to facilitate rapport and support a shared understanding of editorial practices.

Interviews were conducted via Google Meet, lasted on average 57 minutes (range 44–68), and were audio-recorded and transcribed using Google’s automated transcription tool. KB reviewed all transcripts while listening to the recordings to correct errors and ensure accuracy.

### Analysis

Our initial engagement with the transcripts broadly attended to participants’ accounts of publication timelines, pandemic-related disruptions, editorial processes, and leadership. Questions about editorial leadership, challenges and adaptations, communication, and managing publication timelines were particularly helpful in eliciting broader accounts of participants’ editorial practices and responsibilities. Across these accounts, we noted that participants repeatedly described the work they performed to shape scholarship, manage relationships and resources, and sustain journal processes. We therefore focused the present analysis on these recurring accounts of EIC work.

We analyzed the interview transcripts using Braun and Clarke’s six-step thematic analysis approach [23,28,29]. We began by familiarizing ourselves with the data, reading each transcript in full multiple times and noting initial impressions and analytic reflections. Using Dedoose, KB generated initial codes across the dataset, identifying patterns of meaning relevant to the participants’ roles. These codes were then examined in relation to one another to identify broader patterns, yielding candidate themes that described EIC roles. LM reviewed and refined these candidate themes by returning to the transcripts to ensure coherence within themes and distinction between themes. We then defined and named the themes, clarified the scope and boundaries of each role, and selected representative quotes from multiple participants across the dataset. To protect participant confidentiality, we removed participant identifiers from quotations so that readers could not link multiple excerpts to a single participant and construct a more detailed profile of that individual or their journal.

Throughout the analysis, we used several strategies to strengthen our interpretations. Transcripts were verified against the audio recordings, and we engaged repeatedly with the full dataset throughout coding and theme development. The research team met regularly to discuss analytic decisions, consider alternative interpretations, and reflect on how our differing positions within medical education publishing shaped the analysis. Additionally, TLM provided additional insights to team meetings and the writing of the manuscript. Throughout the process, the research team discussed analytic decisions, interrogated emerging patterns of meaning, and reflected on how team members’ perspectives shaped theme development. The proposed themes, their definitions, and illustrative excerpts were also shared with AA for further refinement and critique.

### Reflexivity

This study was informed by the research team’s varied positions within the medical education publishing ecosystem. LM, TLM and AA have held multiple editorial roles in HPE journals, including an EIC role (LM) and Deputy Editor roles (AA, TLM), and all of us have experience as medical education authors. In addition, the research team included junior scholars actively publishing in medical education and HPE journals while navigating time-sensitive promotion and tenure expectations, and who had experienced both manuscript acceptance and rejection. These differing career positions prompted us to examine how time and editorial roles can intersect with career progression.

## Results

Nine EICs, based in five countries on three continents, agreed to participate and were interviewed. Six participants identified as women and three as men. Across the interviews, EICs described their work not as a single, discrete function but as a “whole set of things that we do.” We identified *knowledge developer, proactive communicator, steward of time and energy, nurturer*, and *orchestrator* as five key interrelated roles that spanned the publication lifecycle and shaped how EICs carried out their responsibilities. Together, these roles illustrate how EICs go beyond procedural oversight to actively shape scholarly work, safeguard limited community resources, care for authors and editorial teams, and coordinate complex human and technical systems. These roles were fluid, overlapping, and at times contradictory, reflecting EICs’ efforts to balance intellectual rigor, relational labor, and system constraints in order to sustain the production of scholarly knowledge.

### Knowledge Developer

Participants described the EIC’s role as a knowledge developer. In this role, the EIC viewed shaping the intellectual direction and field-specific boundaries of scholarship as “very important or meaningful.” This role extended beyond selecting or blocking manuscripts for publication or uncritically following reviewer recommendations. Rather, EICs described influencing what counts as legitimate knowledge, situating submissions within the field’s broader conversations, curating scholarship, and actively facilitating conceptual development. As one participant noted: “we wanted to facilitate, you know, the sharing of that information and the sharing of those experiences, for the good of the field.”

Participants also described their knowledge development roles as generative. Several recounted stretching their journals’ scope, creating new article types, or mentoring authors to expand the range of contributions they could submit. For example, one editor launched a rapid-response series to share timely lessons learned during the pandemic, while another commissioned authors to kick-start a new publication type. Participants redirected scholarship which they viewed as valuable, but that did not “meet the bar,” by suggesting authors resubmit under alternative publication types (e.g., innovation reports) that better aligned with the manuscript’s nature and rigor.

As knowledge developers, participants emphasized that this role required constant calibration of standards for manuscript acceptance, which included navigating the tension between maintaining rigor and responding to rapidly evolving priorities. The focus on quality was evident; one participant stated, “my job is to protect the integrity of the knowledge.” The COVID-19 pandemic made this calibration work particularly visible, as EICs confronted both rapidly changing knowledge needs and increased pressure on journal capacity. Another EIC reflected on the “continuous recalibration of what scholarship meant during that time [COVID-19 pandemic]” and how this, in many cases, led to rejecting “very meritorious work” due to page limits, despite the “heartache” of rejecting “fine, fine pieces of scholarship.” These accounts illustrate how the pandemic required EICs to make decisions not only about the quality of individual manuscripts but also about what knowledge was most important to disseminate under rapidly changing conditions and resource-constrained circumstances. In this way, the EICs acted not only as gatekeepers protecting the evidence base, but also actively as judges defining standards of rigor, relevance, and the field’s priorities.

### Proactive Communicator

EICs communicated with a variety of stakeholders, including associate editors, deputy editors, editorial assistants, publishers, sponsoring associations, reviewers, and authors to shape how the journal’s processes and identity were understood. Communication was described as constant, multi-modal, and strategic rather than sporadic. As one participant noted, “Lots and lots of communication. So, it would be written communication, either in [the online journal system] or email. And then frequent [virtual] meetings with the full team or subsets of teams or one-on-one meetings.” These exchanges help EICs maintain continuity across their geographically distributed teams.

Participants described the EIC as responsible for communicating proactively to prevent delays and maintain publication momentum. This involved anticipating breakdowns, monitoring progress, and initiating communication to keep manuscripts and people moving forward. For example, one participant described intervening directly in the production workflow, “I’ll send an email to the production team and say, ‘Hey, I noticed that we sent the accept letter a while ago, but we haven’t seen proofs. Is there something going on with this manuscript?’”

Participants used communication as a problem-solving tool. The pandemic made this problem-solving function more visible. Participants described adapting their communication practices in response to substantial increases in submission volume during this period, implementing coordinated systems to monitor manuscript status and communicate emerging pressures across their teams. For instance, multiple participants adapted their communication practices in response to rising submission volumes. One participant noted, “a X% year-over-year increase in the number of submissions” and another noted, “we literally doubled our submissions.” To manage these pressures, participants implemented coordinated systems to monitor and communicate manuscript status to their team members. Communication was also foundational to cultivating editorial culture and reinforcing journal identity. One EIC described using editorial meetings to discuss, “What are we about? What is our brand? What’s our ethos? What do we want to be known for?” These practices help sustain relationships, foster collegiality, and shape how the journal was perceived internally and externally.

Communication with authors required clarity, attention to tone, and boundary-setting. EICs described balancing reassurance with fairness, especially when authors inquired about timelines. As one participant explained:

So we’ve always had the goal of returning manuscripts within X days. We still do that in 95% of cases, but there is an increasing rate at which papers are lasting longer than that. So there have been a few more queries in recent months, people saying ‘I submitted the paper on this date. Can you give me some guidance?’ I try first of all to alert them to the reality that we’re doing the best we can, that reviewers are harder and harder to come by… So usually, it’s just better to point out for some reassurance that we haven’t lost your paper…and we will get back to you as fast as we possibly can.

EICs also used communication to publicly manage submission trends; one participant described publishing an editorial requesting “please no more of this” in response to a “big pulse of commentaries.” This example demonstrated how communication extended beyond individual interactions to publicly address author behavior and journal direction.

### Steward of Time and Energy

Participants discussed the EIC role as being responsible for safeguarding the limited cognitive and professional resources of reviewers, authors, editors, and themselves. In this role, EICs did not merely manage workload, but they proactively streamlined the editorial process to prevent the resource drain. By prioritizing efforts where they were most warranted, EICs ensured that neither reviewers nor editorial staff were taxed by avoidable editorial inefficiency.

Protecting the limited reviewer pool was a critical aspect of the stewardship role. One editor stated, “My principle is not to waste peer reviewers’ time.” For some, stewardship therefore required editorial restraint in the recruitment and use of reviewers. Another EIC criticized excessive reviewer invitations and argued that editors should be prepared to make decisions without accumulating unnecessary reviews, “there are some times when a paper gets five reviews. The editor should be able to make a decision way before five reviews. This is a waste. Also it’s a major challenge for an author to respond to all of those comments.” For these participants, decisiveness meant that the faster a decision was made, the sooner communal energy could be redistributed to other projects.

Participants framed the first editorial decision (i.e., desk reject) as essential for stewardship. Rapid desk rejection was seen as important to “support the author” so that they can quickly move their manuscript on to another journal. Others stressed that early rejection, when fatal flaws were evident, was a kindness to the author and important for preserving the reviewer pool. One participant noted:

“if you get a review…and it finds a fatal flaw…reject the paper at this point. You don’t need to hold up the author or waste another reviewer’s time…I think this is fairer for the author. They can now move on. It’s also fairer for our community so that resources are not being wasted for no reason.”

This approach positioned desk rejection and limited review cycles not as harsh gatekeeping, but as critical stewardship of reviewers’ limited time and energy.

Stewarding time and energy also extended to the editorial team. Participants described intentionally building systems to prevent bottlenecks in processes and protect against burnout.

Several EICs described proactive check-ins to offer support when their editors appeared potentially overwhelmed, such as reassigning manuscripts as needed. Structural strategies, such as lenient leave policies, were also used to prevent overtaxing editors. By “checking in… making sure they were in a good space” and inviting editors to be “whole people,” EICs maintained team morale.

### Nurturer

Participants acted as nurturers, a role centered on building long-term capacity and scholarly community. In this role, EICs used developmental feedback and relational support to foster the scholarly growth of authors, reviewers, and the editorial team, particularly those early in their careers or navigating challenging circumstances. Without nurturing, participants stated that the publishing ecosystem would weaken; submission and review quality would decline, reviewer pools would shrink, and editorial teams would face attrition. As such, nurturing was viewed as essential to sustaining the field’s capacity.

Cultivating the reviewer and editor pool was a primary focus of the nurturer role. To prevent future personnel shortages, one EIC created a developmental editor program as a structural strategy to mentor emerging editors. Another framed their efforts more expansively, “Investing in reviewers is investing in the field and investing in authors.” This relational labor was often invisible, yet EICs saw it as essential for securing the long-term viability of the publishing ecosystem. Across participants, nurturing was not described as incidental kindness, but a deliberate orientation towards sustaining the journal’s human infrastructure and strengthening the field through relational investments.

Nurturing, in the form of developmental support, was especially directed toward junior scholars. EICs described identifying early-career authors and intentionally offering additional guidance. One explained, “if we notice that we have PhD student authors or medical student authors, or someone that we notice is junior, we will kind of flag that one, to maybe give them a little bit more feedback.” Another articulated that their journal’s ethos is “very much to nurture, not just to receive, but to nurture new material and authors’ skills.” This developmental stance shaped how EICs rejected manuscripts. One EIC reflected that their journal was “so conscious that authors who come to [journal name] are often first time authors…and you and I both know about how scarring that first experience is when you get rejected.” Thus, nurturing shaped not only editorial decisions, but how they were communicated. Finally, participants described nurturing as involving hands-on manuscript refinement and support for authors facing structural barriers. For example, one editor described providing “a tremendous amount of help with [non] native English speakers. I mean, sometimes there’s more words from me than there is from the writer.”

However, this developmental orientation was not universal. One EIC emphasized that editorial decisions must remain anchored in the rigor and integrity of the scholarship itself, explaining, “I’m not going to say, ‘Well they are students. Okay, it is a crap paper, but you know, I’m going to let them in.’ I’m not going to do that. They want to play in the big adults’ pool, then they need to do that.” This contrasting perspective delineates a boundary of the nurturer role. For this EIC, developmental support could facilitate participation in scholarly publishing, but should not lower the standards by which scholarship itself is judged.

### Orchestrator

Participants described their role as ensuring the overall functioning of the publication process. EICs were responsible for “keeping the machine rolling.” These coordinating activities included continuously monitoring manuscript progress, managing handoffs across the editorial team, and intervening when delays, reviewer shortages, or technical issues emerged. For example, EICs described tracking each manuscript and its timeline to identify bottlenecks, and reviewing editors’ average turnaround times “to calibrate and figure out where the holdup is.” When a manuscript was held up, the EIC intervened: “I will go and check the status in the system and see what’s happening. If there is a problem – if an editor does seem to be sitting on it – I will send a rocket to them.”

This role involved multiple tracking systems and regular review of workflow data. One EIC mentioned “tracking all of it” by using editorial management software reports to monitor peer review, spreadsheets to track manuscript production, and documents to keep tabs on assignments made to their editorial team. Detailed tracking provided a “finger on the pulse of manuscripts that might be in trouble.” By synthesizing data points, EICs identified gaps before they became significant publication delays. Reviewer recruitment was a common “slowdown hotspot.” When manuscripts stalled because reviewers declined invitations or could not be identified, EICs relied on personal networks and individual requests to secure reviewers. One EIC noted, “We went to 22 reviewers on a paper… We begged, we asked favors. And I felt a real responsibility to do that.” The orchestrator role acted as a manual override to the automated system, preventing manuscripts from idling indefinitely.

## Discussion

This study provides an empirical account of how EICs understand and enact their roles. Rather than describing EIC roles as a set of discrete tasks, participants articulated five interrelated roles that together shape the publication process. EICs described simultaneously shaping knowledge (*knowledge developer*), coordinating communication across stakeholders (*proactive communicator*), safeguarding finite communal resources (*steward of time and energy*), building capacity within the scholarly community (*nurturer*), and maintaining workflow continuity (*orchestrator*). These roles were not enacted sequentially, but as overlapping, and at times competing responsibilities. In this way, the EICs described roles that extended beyond procedural oversight to encompass ongoing, socially organized work that requires constant calibration of priorities, relationships, and constraints. By making these roles visible, our findings help open the “black box” [2] of academic publishing and highlight the human, relational, and infrastructural work through which scholarly journals are managed and knowledge is produced.

This expanded view of roles extends prior work that has primarily characterized editors as gatekeepers or academic developers emphasizing their roles in filtering or improving manuscripts [13,30]. While these functions were described in our data, particularly within the knowledge developer and nurturer roles, our findings reveal a broader, more integrated set of responsibilities. Participants described not only evaluating and selecting manuscripts, but actively shaping their trajectory, redistributing resources, supporting contributors, and intervening in system breakdowns. Recognizing this complexity can help move discussions of editorial work beyond reductive binaries and toward a more nuanced understanding of how knowledge is both filtered and cultivated within academic publishing.

A key contribution of this study is making visible the largely invisible labor that underpins academic publishing. Invisible labor refers to work that is “often overlooked, ignored, or devalued.” [31 (p6)] Participants described continuously “keeping the machine rolling” through extensive behind-the-scenes work, including monitoring manuscript progress, troubleshooting delays, recruiting reviewers, supporting editorial team members, and fostering relationships. These activities are rarely captured in formal metrics or visible within manuscript tracking systems and likely are overlooked. Yet, our participants described them as essential to sustaining the publication process. This research provides future EICs with insight into the role and alerts authors to the less-apparent work that EICs undertake. What may appear to authors as delay or opacity often reflects ongoing, intensive, but invisible efforts to coordinate people and processes within a constrained system. Making this labor apparent helps remove publishing opacity and reframes the work of EICs as relational and administrative rather than procedural.

Reviewer scarcity is not unique to our participants. In an editorial entitled “Where have all the reviewers gone?” Ellaway directly described the problem of dwindling resources within academic publishing [32]. Our findings echo and extend this concern by highlighting the central role of editorial stewardship within a resource-constrained system that has been described as suffering from reviewer fatigue [33]. Academic publishing relies heavily on volunteer labor, particularly from peer reviewers, whose time, generosity, and expertise are finite. Prior research has examined the reasons manuscripts are desk rejected [11]. Our findings offer a complementary perspective by showing how EICs understood desk rejection as an act of stewardship: a means of conserving limited reviewer resources and avoiding unnecessary demands on reviewers and authors. Participants described a constant need to balance competing commitments of supporting authors through developmental feedback while simultaneously protecting the reviewer pool from running dry. Decisions such as limiting reviewer invitations, increasing the number of desk rejections, or curtailing multiple rounds of review were not framed simply as efficiency measures, but as ethical acts of stewardship for the health of the field.

This tension exposes a structural challenge in scholarly publishing, such that efforts to enhance author development and inclusivity may increase demands on an already strained system. It also highlights a fundamental friction between two core editorial roles—the *nurturer* and the *steward*, where investing in the development of individual manuscripts may come at the expense of collective sustainability. Addressing this tension will require field-level attention to how responsibility for author development is distributed, how reviewer labor is recognized and supported, and how publishing systems can better align expectations for rigor, inclusivity, and sustainability without over-relying on finite volunteer resources.

Our findings also have implications for preparing and supporting current and future EICs. There is no formal pathway to becoming an EIC; editorial leadership is largely learned through experience [15]. While publishers and professional organizations have begun to offer targeted training for EICs (e.g., webinars on peer review or editorial decision-making) [34,35], these efforts tend to focus on discrete tasks rather than the integrated and relational nature of the role. Additionally, there are entry-level educational opportunities (e.g., editorial internships, associate editor roles) [36,37], but EIC education is notably absent. The interconnected framework of roles identified in this study may offer a more comprehensive foundation for EIC training by highlighting the need to develop skills in communication, systems thinking, relational leadership, and resource stewardship alongside traditional editorial competencies.

### Limitations

This study has several important limitations. First, our sample included nine English-language medical education journal EICs, which may limit the transferability of our findings to other disciplines, publishing contexts, or language settings. Second, we focused on EIC perspectives, and the interviews provided limited insight into the role of editorial boards, professional societies, and publishers in journal governance and oversight. Incorporating the perspectives of deputy and associate editors, editorial staff, and other stakeholders would provide a more comprehensive understanding of how editorial responsibilities and decision making are distributed and enacted across journal structures. Finally, these interviews were originally conducted as part of a broader investigation of publication timelines, particularly in the context of COVID-19. This context may have made particular dimensions of editorial work, such as managing delays, communicating with stakeholders, and stewarding limited resources, especially salient in participants’ accounts, while other dimensions received less attention. Although participants repeatedly described broader aspects of their work as EICs, the focus of the original interview guide may have shaped which aspects of the work were most prominent in their interviews. While editorial roles emerged as a primary finding, a study specifically designed to examine these roles may have yielded additional roles or alternative emphases.

## Conclusion

These findings suggest that EIC work can extend beyond procedural oversight. The participants we interviewed here described actively shaping disciplinary knowledge, coordinating complex human and technical systems, stewarding limited communal resources, supporting scholars’ development, and intervening when disruptions threaten the publication process.

## Disclaimers

The opinions and assertions expressed herein are those of the author(s) and do not reflect the official policy or position of the Uniformed Services University of the Health Sciences or the Department of War.

The opinions and assertions expressed herein are those of the author(s) and do not necessarily reflect the official policy or position of the Henry M. Jackson Foundation for the Advancement of Military Medicine, Inc.

## Additional File

The additional file for this article can be found as follows:

10.5334/pme.3277.s1 Supplementary Material. Interview guide.
