# Supplementary material for: “It’s Like a Whole Set of Things That We Do”: A Qualitative Study of the Roles of Journal Editors-in-Chief
Source: Perspect Med Educ. 2026 Oct 15;15(1):1085–94. doi: 10.5334/pme.3277 (PMC13674542; doi:10.5334/pme.3277)
Supplement: Supplementary file 1 [file pme-15-1-3277-s1.pdf]

Interview guide for “It’s like a whole set of things that we do”: A qualitative study of the roles of journal editors-in-chief by Maggio, Artino, Costello, Martin, Ma, and Brown.

1. To start off, my first question is really broad. So, when you think back to the pandemic, what would be 3 words that describe what it was like during that first year?
    - Ask to explain why those words.

2. Now that you are back in that headspace, more specifically what was your experience as an editor during 2020-2021 time.
    - In what ways do you think COVID influenced your editorial leadership?
    - What would you say were the most significant challenges?
    - How did your journal adapt to the pandemic?
    - Can you tell me a story about one of the challenges you experienced?
    - Were there any unexpected positive things during this time period?
    - How did you rally your editors?
    - In what ways if any did you reach out to authors beyond usual editorial communications?

3. Our data, and data from other fields, indicates that the time from submission to publication were impacted during COVID. What factors do you think contributed?
    - Reviewer
    - Author
    - Editor
    - Did you observe any trends among reviewers or authors regarding gender, race/ethnicity, or X?
    - Beyond the Author, Reviewer, and Editor are there other external or internal factors that would have shaped this? For example, did your journal switch editorial platforms or publishers?

4. At your journal in what ways if any do you track the timeline of publications?
    - At what point might you get concerned about an articles time frame?
    - If you notice a slow down, what do you do?
        i. Did you communicate with the authors?
        ii. Engage with editors?
    - In what ways did the peer review process impact the timeline?
    - Are there any factors like publication type or the status of an author for example a PhD student or an early career researcher that might impact how you consider the timeframe?

5. You are also an author. What has your experience been with time to publication?
    - Has it impacted your career in anyway?

- Does a sense of journal's timeline influence where you submit?
- What do you do if you have what you would consider a stuck paper?
- As an author what would you consider to be an unreasonable amount of time to publication?

6. Is there anything else you want to share about factors contributing to the longer publication timeline or your editorial experience during COVID-19?

7. COVID wasn't the only thing happening during the time period. During this timeframe there were many protests. For example, in Hong Kong there were umbrella democracy protests, wildfires in Australia, and in the US the black lives matter movement. These social events may have shifted people's ability or desire to work, so we wanted to know if you think that the social events during this timeframe shape your editorial experience?
    - If yes, how?
    - Do you think world events affected the publication timeline for authors?
    - Do you think these events shaped research topics submitted to your journal?

8. Looking to the future, what do you think editors can do to facilitate the time to publication?
    - What do you think is the role of communication?
    - Many people in our field have lamented the rise in requests for peer review.
    - Do you think there is a role for journals to work together?
    - In what ways if any do you think we would be prepared for another global emergency?

9. What is your advice for an author who's paper seems to be taking a long time?

10. How about advice for an editor who has a paper stuck in their queue?

11. To wrap up, I just have a few quick demographic questions.
    - How would you describe your nationality?
    - How would you describe your race or ethnicity?
    - How would you describe your gender?
    - Are you a parent or caregiver for family members? If yes, how old were your kids when COVID started?
